# Sustained Reduction of Dystonic Tremor and Pain after Cannabis Oil Administration and Physiotherapy in Thalamic Ischemia: A One-Year Case Report

**DOI:** 10.2174/011570159X414414251122103253

**Published:** 2026-01-26

**Authors:** Enrico Buccheri, Salvatore Caramma, Rita Chiaramonte, Michele Vecchio

**Affiliations:** 1 Department of Biomedical and Biotechnological Sciences, Section of Pharmacology, University of Catania, Catania, Italy;; 2 Department of Anestesiology, Azienda Ospedaliero-Universitaria Policlinico Vittorio Emanuele, Catania, Italy;; 3 Rehabilitation Unit, AOU Policlinico Rodolico, Catania, Italy;

**Keywords:** Dystonic tremor, stroke, pain, cannabis oil, rehabilitation, physiotherapy, therapeutic exercise, motor functions

## Abstract

**Introduction:**

Post-stroke tremor and post-stroke thalamic pain (PS-TP) are common and often refractory conditions that significantly impact patients’ quality of life. Conventional pharmacotherapy frequently provides inadequate relief, while cannabis has shown potential for managing movement disorders and pain; however, evidence supporting its efficacy remains limited. On the other hand, physiotherapy is well-documented as an effective therapeutic intervention.

**Case Presentation:**

This case report aimed to evaluate the combined effects of cannabis oil and physiotherapy on dystonic-tremor and PS-TP in a female subject with a history of thalamic ischemia. The patient was monitored over a 1-year follow-up period with assessments focused on pain intensity, tremor severity, and overall functional improvements. After twelve months of treatment, the patient demonstrated a 60% reduction in pain and a 56.88% reduction in tremor severity, accompanied by enhanced motor function. Furthermore, quality of life improved significantly, with a 27.6% increase in the mental component and a 45.46% increase in the motor component. No serious adverse effects were reported during the treatment period.

**Conclusion:**

This case report highlights the potential benefits of combining cannabis oil with physiotherapy for managing post-stroke dystonic tremor and PS-TP. The sustained efficacy of this treatment combination over a prolonged period could constitute a therapeutic novelty and an important advancement in the management of these conditions. These findings suggest the need for further research with larger cohorts and studies of higher methodological rigor to establish the efficacy and safety of this therapeutic approach.

## 1 INTRODUCTION

Cerebrovascular diseases are among the most common causes of secondary movement disorders, with thalamic ischemia being particularly associated with tremors and dyskinesias, with a prevalence of about 37% [1]. Post-stroke tremor and post-stroke thalamic pain (PS-TP) are often refractory to pharmacotherapy [2, 3], posing a significant challenge in clinical management.

For instance, botulinum toxin has been employed in the treatment of tremor; however, the available literature on this application remains limited and lacks sufficient robustness [4].

The use of therapeutic cannabis in the treatment of movement disorders remains an area of emerging research. Current evidence supporting its efficacy is primarily derived from preclinical studies [5, 6], case series [7, 8], and clinical studies [9, 10], of which only small numbers are randomized controlled trials (RCTs) [11-13]. Consequently, cannabis is not currently recognized as a conventional pharmacological treatment option [11]. Similarly, PS-TP is typically managed with neuropathic pain medications, which may provide only partial relief in some patients [3]. Conversely, physiotherapy has been well-documented as an effective therapeutic intervention for both pain [14] and movement disorders [15, 16]. In other areas, such as fibromyalgia pain, physiotherapy can be beneficial, and when combined with cannabis, the effects can be even better [17, 18].

Rehabilitation approaches, including sensorimotor training and neuroplasticity-based interventions, have shown promise in improving functional outcomes in patients with post-stroke complications, such as walking velocity and the use of a hemiparetic extremity, with the focus on motor-skills learning as a central role in new stroke rehabilitation designs [19].

Given the limited efficacy of pharmacotherapy in addressing both post-stroke tremor and PS-TP, there is growing interest in multimodal treatment strategies.

This case report explores the potential benefits of combining cannabis oil and physiotherapy in the management of a patient with thalamic ischemia-related dystonic tremor and PS-TP. By documenting the therapeutic approach and patient response over a one-year period, this report aims to contribute to the understanding of alternative treatment strategies for these challenging post-stroke complications.

## 2 CASE PRESENTATION AND NARRATIVE REVIEW

This case report was prepared in accordance with the CARE guidelines. The principles outlined in the Declaration of Helsinki were followed in the management and reporting of this case involving a human subject.

A 30-year-old female patient with a history of thalamic ischemia in 2006 was referred to the Pain Therapy Unit of University Hospital Policlinico-San Marco of Catania in May 2022. The patient provided written informed consent for both the treatment and the evaluations conducted during the follow-up period.

The patient was affected by hyperalgesia on the left side, predominantly affecting the upper limb, with both proximal and distal involvement, as well as a continuous tremor in the same region. The ischemic lesion affected the internal capsule and the thalamus on the right side, particularly involving the anterolateral nuclei. The patient’s symptoms included dystonic tremor, mild hypoesthesia, hyperalgesia, allodynia, and hemiparesis.

More than 5 types of treatments (different types of physiotherapy, different antiepileptic drugs, different opioid drugs) for both dystonic tremor and pain had been attempted over several years, but none have provided significant benefit.

The patient was prescribed therapeutic cannabis in the form of Bediol oil, authorized in Italy as a component of medical cannabis for chronic pain treatment. Bediol oil, produced by Bedocran, is extracted from the *Cannabis sativa* L. strain and contains 8% cannabidiol (CBD) and 6.5% tetrahydrocannabinol (THC), in addition to terpenes, such as myrcene, α-2-pinene, and β-caryophyllene. The oil was administered to the patient at a dose of 12 drops twice a day.

This treatment was combined with physiotherapy, which was administered three times per week and included manual therapy performed in three-month cycles, totaling two cycles over 12 months. Additionally, a self-guided rehabilitation protocol for muscle strengthening was implemented (Table **S1**).

The rehabilitation protocol consisted of sequential stretching, aerobic exercises, and progressive-load calisthenic exercises, partly derived from the widely cited 5BX plan protocol [20]. The program was performed for 35-40 minutes, three times a week. This protocol was selected because evidence demonstrated the effectiveness of various rehabilitation training programs in stroke patients [21].

Baseline assessments were conducted using the rating scales listed in Table **1**.

For each scale, measurements were obtained at baseline (T0, prior to treatment initiation), at 1 month (T1), and at 6 months (T6), following the start of the combined treatment. Percentage changes from baseline to T1 and from baseline to T6 were also calculated to quantify treatment-related improvements.

The data presented in Table **1** show a progressive improvement across all rating scales from baseline (T0) through 1 month (T1), 6 months (T2), and 12 months (T3) following the combined treatment with cannabis and physiotherapy. The SF-12 Mental Component Summary (MCS) score improved by 27.6% at T3, indicating enhanced mental health-related quality of life, while the SF-12 Physical Component Summary (PCS) score increased by 45.46%, reflecting improved physical health. Pain, as measured by the Numeric Rating Scale (NRS), decreased by 60% at T3, indicating significant pain relief. Additionally, the Fahn-Tolosa-Marin Tremor Rating Scale (FTM-TRS) showed a 56.88% reduction in tremor severity at T3. These findings suggested sustained and clinically meaningful benefits from the combined therapy over the 12-month period.

The patient demonstrated good adherence to cannabis treatment, consistently maintaining daily intake throughout the 12-month period. Adherence to self-guided physiotherapy and manual therapy was moderate, with a total of 6 months of treatment completed over the 12 months (2 months of treatment and 2 months of non-treatment alternating). The patient showed significant improvement across all three rating scales, with benefits persisting up to 12 months from the start of the treatment, as shown in Table **1**.

This case report falls within the broader topic of using cannabis for the treatment of pain and tremor. While several studies have investigated cannabis for pain management [22], research on its effects on tremor remains limited.

To explore the existing literature, a search was conducted in major databases, including PubMed, Scopus and Web of Science, using the search terms “tremor” and “cannabis”, to ensure a comprehensive retrieval of relevant studies. Only English-language articles of any type (case report, clinical trial, systematic review, meta-analysis) were included. The initial search yielded 96 articles, which were then screened by title and abstract, resulting in 12 articles selected for further analysis. (Table **2**). The methodology of analysis of these articles was based on showing the effects of cannabis on tremor in various pathologies (Parkinson's, essential tremor, Huntington's disease) to better understand its possible potential.

## 3 DISCUSSION

The findings of this study suggest a potentially relevant effect of the combined use of cannabis and physiotherapy on pain control, dystonic tremor reduction, and overall quality of life. However, evidence from a single case cannot be generalized. Further research involving larger patient cohorts, extended follow-up periods, and direct comparisons with standard pharmacological treatments is warranted to substantiate these preliminary observations.

On the other hand, there is substantial evidence supporting the efficacy of physiotherapy and therapeutic exercise in both pain management [14] and the treatment of movement disorders [15, 16].

Various formulations of medical cannabis have demonstrated some beneficial effects on pain in several studies reported in the literature [35, 36], although the available evidence remains limited with regard to post-stroke pain [37, 38].

Conversely, the exact mechanism by which cannabis affects tremor and dystonia has not yet been fully explained. One hypothesis suggests that cannabinoid receptor type 1 (CB1) agonists reduce hyperactivity in the globus pallidus interna (GPi) and improve dystonia by promoting gamma-aminobutyric acid (GABA) reuptake [11]. The GPi and substantia nigra pars reticulata are rich in CB1 receptors located presynaptically on GABAergic terminals. Activation of these receptors has been shown to modulate GABA release, suggesting a neuromodulatory role of cannabinoids in these regions. However, the exact impact of CB1 receptor activation on GABA dynamics within the GPi remains a subject of ongoing research [39, 40]. Modulation of other receptors by cannabis, such as transient receptor potential vanilloid 1 (TRPV1), may also have substantial impacts on neuronal activity and motor control [41].

Furthermore, cannabinoid receptors and endocannabinoids are highly expressed in brain regions controlling movement, such as the basal ganglia and cerebellum. Endogenous, plant-derived, and synthetic cannabinoids modulate motor activity, at least in part, through effects on classical neurotransmitters, including dopamine, GABA, and glutamate, in the basal ganglia. Alterations in endocannabinoid signaling have been observed in both human movement disorders and corresponding animal models [42]. Recently, these findings have been extended by the identification of additional mechanisms influencing motor function (*e.g*., interactions with adenosine receptors in Huntington’s disease) [43], the discovery of new cannabinoids [44], and the visualization of the cannabinoid system in healthy individuals and patients using molecular imaging [45].

Despite these theoretical mechanisms, clinical studies have yielded mixed results regarding the efficacy of cannabinoids in treating dystonia. For instance, a randomized controlled trial investigating the use of dronabinol, a CB1 agonist, in patients with cervical dystonia found no significant therapeutic benefit [39], just as no significant results were found in an RCT on the use of cannabis in the treatment of tremor in patients with multiple sclerosis [46]. For example, the use of 300 mg of CBD had a significant effect on reducing tremor and anxiety in an RCT on Parkinson's patients [47], as well as anxiety and anxiety-induced tremor in patients affected by Parkinson's could be reduced in the CBD group, compared to the placebo in another RCT [48].

Studies that have generally investigated the effects of cannabis on movement disorders, as summarized in Table **2**, with some reporting the efficacy of cannabis in managing tremor and other movement disorders [24, 26, 28-30, 33, 34], consistently emphasize the need for further RCTs with larger patient samples. This is particularly important given the limited number of studies on the topic and their generally low methodological quality [23-34]. However, the systematic review by Akinyemi *et al*. [28] reported the excellent use of cannabis in a large number of patients with tremor (361) and suffering from Huntington's disease, thus making it necessary to investigate the real effectiveness of cannabis.

Finally, a recent review has highlighted the heterogeneity of evidence on the effects of cannabis in movement disorders, underscoring the need for further clinical studies to better clarify its therapeutic benefits and long-term adverse effects [49].

The limitations of this study are inherent to its design as a case report, and therefore relate to the effects observed from the combination of cannabis and physiotherapy in a single patient. Further well-structured studies are required to clearly establish the combined efficacy of cannabis and physiotherapy in patients suffering from pain and dystonic tremor.

## CONCLUSION

This case report highlights the potential importance of combining medical cannabis oil with physiotherapy in managing pain and tremor in patients with thalamic ischemia sequelae. The marked improvement in both pain and tremor (each reduced by more than 50%), together with the observed enhancement in quality of life, underscores the need for further research in larger cohorts and studies with higher levels of evidence to assess the potential role of cannabis, in combination with physiotherapy, in managing movement disorders and pain. Given the low level of evidence currently available in the literature and the near absence of evaluations of the combined effect of cannabis and physiotherapy, long-term randomized controlled trials are required. These trials should compare this protocol with established gold standards and incorporate extended follow-up. Such studies represent a key research priority to strengthen the evidence base for the management of patients with pain and movement disorders.

## Figures and Tables

**Table 1:** Percentage improvement on rating scales at T1, T2, and T3 compared to baseline (T0).

**Rating Scales**	**T0 (Baseline)**	**T1 (1 Month)**	**% (T0-T1)**	**T2 (6 Months)**	**% (T0-T2)**	**T3 (12 Months)**	**% (T0-T3)**
**SF-12 MCS**	36.05	38.29	6.2%	42.47	17.7%	46.03	27.6%
**SF-12 PCS**	31.03	50.39	38.74%	52.10	40.44%	56.93	45.46%
**NRS**	10	6	40%	5	50%	4	60%
**FTM-TRS**	30.50	14.5	52.46%	14.50	52.46%	13.15	56.88%

**Abbreviations:** Short Form Health Survey-12 (SF-12); Mental Component Score (MCS); Physical Component Score (PCS); Numeric Rating Scale (NRS); Fahn-Tolosa-Marin Tremor Rating Scale (FTM-TRS).

**Table 2:** Studies considered for the literature review.

**Study**	**Type of Study**	**Aim**	**Outcomes**	**Results/Conclusion**
Arjmand *et al*. (2015) [23]	Narrative review	To describe the possible effects and the molecular mechanism of cannabis to reduce tremors in Parkinson’s disease (PD), multiple sclerosis (MS), and Huntington’s disease (HD)	- Tremor in PD - Tremor in MS - Tremor in HD - Ataxia	- Results actually did not reach statistical significance
Farzaei *et al*. (2017) [24]	Narrative review	To review the clinical evidence related to medicinal plants in the treatment of MS	- Tremor and spasticity - Long-term use of cannabis - Pharmacological mechanisms of cannabis	- *Cannabis sativa* presented good clinical evidence in the treatment of MS symptoms
Nielsen *et al*. (2018) [25]	Systematic review	Review of the review articles showing the safety and effectiveness of cannabis in MS	- Disability and disability progression - Pain - Spasticity - Bladder function - Ataxia and tremor - Sleep quality - Quality of life - Adverse effects	- 11 systematic reviews were included - 5 reviews showed sufficient evidence that cannabinoids may be effective for the treatment of pain and/or spasticity in MS - Mild to moderate adverse effects (dizziness, dry mouth, euphoria, diarrhoea, and difficulty concentrating) - Non-significant results on efficacy in the case of tremor
Bougea *et al*. (2020) [26]	Systematic review	1) To evaluate the efficacy of cannabis 2) To explore the factors interfering with cannabis use in PD	- Tremor - Motor symptoms - Unified Parkinson's Disease Rating Scale (UPDRS) - Pain - Sleep quality - Quality of life - Depression, anxiety - Levodopa-induced dyskinesias - Cognitive impairment	- 866 patients were considered - Positive effects on motor and non-motor symptoms described in uncontrolled studies - Only one RCT found a reduction in levodopa-induced dyskinesias - One RCT reported a reduction in anxiety and tremor - The other three RCTs reported no effect on motor/non-motor symptoms - Actually, the use of cannabis in PD is not recommended
Fiani *et al*. (2020) [27]	Narrative review	To show the efficacy of CBD in the management and treatment of various neurological disorders	- Pain - Tremor - Tremor and motor symptoms in PD - Anxiety and related disorders - Outcomes of epilepsy - Spasticity and other symptoms in MS	- Few studies still show efficacy for tremor in PD, MS, and essential tremor (in this case, only in animal models) - In the United States, the use of cannabis has been approved for the treatment of spasticity in MS, as well as in Dravet and Lennox-Gastaut syndromes
Akinyemi *et al*. (2020) [28]	Systematic review	To evaluate the effects of cannabis on symptoms of movement disorders with a focus on HD	- Choreic movements - Chorea - Tremor - Psychomotor decline - Cognitive impairment - Sleep quality - Depression	- 2014 patients were considered (361 for the evaluation of tremors) - Strong evidence in the 22 studies reviewed for a significant improvement in the neurologic symptoms, such as spasms, tremors, spasticity, chorea, and quality of sleep with cannabis treatment - Significant improvement in tremors and rigidity
Oikonomou *et al*. (2022) [29]	Systematic review	Systematic review of RCT on the use of cannabis in movement disorders	- UPDRS for PD symptoms - Unified Huntington's Disease Rating Scale (UHDRS) for HD symptoms	- 311 patients were considered - Seven RCTs on PD used different cannabis formulations - Anxiety and anxiety-induced tremor could be reduced in the cannabidiol group in one RCT - In two RCTs with Tourette syndrome, an improvement in tics was reported - From three RCTs on Huntington's disease, only one reported symptom relief using nabilone
Urbi *et al*. (2022) [30]	Systematic review and meta-analysis	To report the effects of cannabis in PD	- UPDRS - Levodopa-induced dyskinesias - Tremor - Pain - Improvement in sleep - Quality of life	- 1388 patients were considered - Potential benefit in the alleviation of tremors, anxiety, pain, and improvement of sleep quality and quality of life in patients with PD
Pourmohammadi *et al*. (2022) [31]	Systematic review	A review of pharmacological treatment studies on tremor in MS to define the treatment recommendations	- Tremor evaluated with different rating scales	- 1516 patients were considered - The application of cannabis to treat tremor in MS could not be recommended due to inconclusive therapeutic effects and several side effects
Haddad *et al*. (2022) [32]	Narrative review	To investigate the effects of cannabis on symptoms in MS patients	- Disability and disability progression - Pain - Spasticity - Bladder function - Ataxia and tremor - Sleep quality - Quality of life - Adverse effects	- No effect on tremor of nabiximols or oral cannabinoids has been reported while being effective for spasticity and pain - Mild to moderate adverse effects (dizziness, dry mouth, euphoria, diarrhoea, and difficulty concentrating)
Aladeen *et al*. (2023) [33]	Clinical trial	A chart review to explore the impact of cannabis on the treatment of patients with PD	- Dystonia - Pain - Spasticity - Lack of appetite - Dyskinesia - Tremor	- 69 patients were considered - Cannabis may improve motor and nonmotor symptoms in PD
Issa *et al*. (2025) [34]	Case report	To present a treatment with *Cannabis sativa* oil of a 77-year-old man diagnosed 22 years ago with PD in an advanced stage, with bradykinesia, tremor and rigidity, and inability to maintain an upright position and walk	- Motor symptoms of PD - Non-motor symptoms of PD	- After the treatment, the patient was able to walk around the house - Significant improvement in non-motor symptoms

## Data Availability

All the data and supporting information are provided within the article.

## LIST OF ABBREVIATIONS

CB1: Cannabinoid Receptor Type 1
CBD: Cannabidiol
FTM-TRS: Fahn-Tolosa-Marin Tremor Rating Scale
GABA: Gamma-Aminobutyric Acid
GPi: Globus Pallidus Interna
HD: Huntington’s Disease
MCS: Mental Component Score
MS: Multiple Sclerosis
NRS: Numeric Rating Scale
PCS: Physical Component Score
PD: Parkinson’s Disease
PS-TP: Post-Stroke Thalamic Pain
RCT: Randomized Controlled Trial
SF-12: Short Form Health Survey-12
THC: Tetrahydrocannabinol
TRPV1: Transient Receptor Potential Vanilloid 1
UHDRS: Unified Huntington's Disease Rating Scale
UPDRS: Unified Parkinson's Disease Rating Scale

## STANDARDS OF REPORTING

CARE guidelines were followed.
