# Supplementary material for: Sustained Reduction of Dystonic Tremor and Pain after Cannabis Oil Administration and Physiotherapy in Thalamic Ischemia: A One-Year Case Report
Source: Curr Neuropharmacol. 2026 Jan 26;24(6):895–901. doi: 10.2174/011570159X414414251122103253 (PMC13519554; doi:10.2174/011570159X414414251122103253)
Supplement: Supplementary file 1 [file CN-24-6-895_SD1.pdf]

Supplementary Material

Sustained Reduction of Dystonic Tremor and Pain after Cannabis Oil Administration and Physiotherapy in Thalamic Ischemia: A One-Year Case Report

Enrico Buccheri[1], Salvatore Caramma[2], Rita Chiaramonte[1,*] and Michele Vecchio[1,3]

[1]Department of Biomedical and Biotechnological Sciences, Section of Pharmacology, University of Catania, Catania, Italy; [2]Azienda Ospedaliero-Universitaria Policlinico Vittorio Emanuele Anestesiology, Catania, Italy; [3]Rehabilitation Unit, "AOU Policlinico Rodolico", Catania, Italy

Table S1. Self-guided rehabilitation protocol.

Exercises	Description	Progression
**Warm-up** (5 minutes)		
Walk	Walk at slow speed	Increase speed below fatigue threshold
Active stretching	Active torsional stretching of the muscles (paraspinal muscles, upper and lower limbs)	-
**Aerobic exercises** (10 minutes)		
Running	Run with correct breathing and correct rhythm of the upper limbs	Increase speed
**Progressive load exercises (**15 – 20 minutes)		
Crunches	Flexion of the trunk forward in a supine position with bent knees	Insert torsional component for the oblique muscles and increase the number of set
Squat	Bilateral squat soft	Monolateral squat
Back extension	Trunk extension in the supine position	Put the trunk progressively outside of a bench
Plank	In a prone position, rest your hands with elbows extended or rest your forearms with elbows flexed, trying to maintain the straight line between head-back-lower limbs	Increase the period of the exercise
Push-up	From the prone position with elbows flexed, fully extend the elbows (possibility of resting your knees on the ground)	Increase the number of set
Theraband exercises	For the upper limbs (in anterior flexion, abduction, internal and external rotation), for the lower limbs (adductors, abductors, flexors, extensors)	Increase the number of set and the resistance of the theraband
**Cool-down (**5 minutes)		
Static stretching	Static stretching of the muscles of the main body segments (spine, shoulder, elbow, wrist, hip, knee and ankle)	-

The *self-guided rehabilitation protocol* is based on sequential stretching, aerobic exercises and progressive load calisthenic exercises, the latter partially derived from the 5BX plan protocol, which is extensively cited in the literature [5BX Plan for Physical Fitness (PDF) (3rd ed.). Royal Canadian Air Force. 1975]. The protocol was performed for 35 – 40 minutes, 3 times a week. Different rehabilitation training protocols have proven to be effective in stroke patients [Vecchio M, Gracies JM, Panza F, Fortunato F, Vitaliti G, Malaguarnera G et al. Change in Coefficient of Fatigability Following Rapid, Repetitive Movement Training in Post-Stroke Spastic Paresis: A Prospective Open-Label Observational Study. J Stroke Cerebrovasc Dis. 2017; 26(11): 2536-2540].

1875-6190/26 $65.00+.00 **© 2026 Bentham Science Publishers**
